# P-1000. Evaluating The Efficacy of Oral Vancomycin Primary Prophylaxis to Prevent Clostridioides difficile Infections in Heart Transplant Recipients

**DOI:** 10.1093/ofid/ofaf695.1198

**Published:** 2026-01-11

**Authors:** Adam Grunseich, Reshma George, Patricia Saunders-Hao, Hind El Soufi, Vidal Luchana, Esther Benamu, Rubab Sohail, Pranisha Gautam-Goyal, Sumeet Jain

**Affiliations:** Northwell - Long Island Jewish Medical Center, New Hyde Park, New York; Donald and Barbara Zucker School of Medicine at Hofstra/Northwell, Dayton and Karen Brown Division of Infectious Diseases, New Hyde Park, New York; North Shore University Hospital, Bayside, NY; Zucker School of Medicine at Hofstra/Northwell, Manhasset, New York; Long Island Jewish Medical Center, New Hyde Park, New York; Transplant Institute, Northwell Health, Manhasset, New York; Northwell Health, New Hyde Park, New York; Zucker School of Medicine at Hofstra/Northwell, Manhasset, New York; North Shore University Hospital, Bayside, NY

## Abstract

**Background:**

*Clostridioides difficile* is a common cause of hospital-acquired diarrheal infections and immunocompromised patients, including heart transplant patients, are vulnerable to severe *Clostridioides difficile* infections (CDI). Primary prophylaxis of CDI with oral vancomycin can be utilized in these patients but data regarding efficacy is limited.

**Methods:**

This was an IRB-approved retrospective observational chart review conducted at North Shore University Hospital evaluating the efficacy of oral vancomycin for primary prophylaxis of CDI in heart transplant recipients admitted between January 2018 and December 2023. The study compared patients who received oral vancomycin prophylaxis (OVP) for CDI with those who did not, based on a protocol change in January 2022. Patients with a history of CDI were excluded. The primary objective was to compare the development of CDI within one-year post-transplant between patients who received OVP and those who did not. The secondary objective was the incidence of vancomycin-resistant *Enterococcus* infections within one-year post-transplant. Statistical analysis involved Fisher’s exact test for categorical data and Wilcoxon rank-sum test for continuous data.

**Results:**

The study included 122 patients. Of these, 44 (36%) did not receive OVP, while 78 (64%) did. For the primary outcome, 2 patients developed CDI within one-year post-transplant, both of whom had not received prophylaxis, but this difference was not statistically significant. These patients developed CDI within 24 days and 39 days respectively. For the secondary outcome, 8 patients developed VRE within one-year post-transplant of which 63% (n=5) received prophylaxis. There was no significant difference in VRE development between those who did and did not receive OVP (6.4% vs. 6.8%, respectively).

**Conclusion:**

This study did not show a benefit for OVP in the prevention of CDI in heart transplant recipients without a history of CDI. However, these results have limitations, primarily due to the low incidence of CDI and the small sample size. Further research is needed to assess the efficacy of oral vancomycin primary prophylaxis in heart and other solid organ transplant recipients.

**Disclosures:**

All Authors: No reported disclosures

